# Ageing, women's health and personalised medicine: Advancing artificial intelligence and therapeutics through the blueprint theory paradigm

**DOI:** 10.1113/EP092854

**Published:** 2026-10-12

**Authors:** Rosie Richardson, Tina Woods, Nikodem Grzesiak, Annalisa Jenkins, Carina C. Kern

**Affiliations:** ^1^ LinkGevity, Babraham Research Campus Cambridge UK; ^2^ Collider Health London UK; ^3^ Genomics England London UK

## 1 INTRODUCTION

Women's health, particularly in the context of ageing, has historically been under‐represented in clinical research, leaving critical gaps in knowledge and limiting the development of targeted therapies. Despite clear physiological differences, such as sex‐based variations in pharmacokinetics and pharmacodynamics, women were often actively excluded from clinical trials until the 1990s (Mastroianni et al., 1994). Consequently, many ageing‐related conditions unique to women remain under‐recognised and undertreated.

For example, menopausal osteoporosis was officially recognised by the World Health Organization (WHO) only in 1994, decades after its significant impact on women's health had already been established (World Health Organization, 1994). Yet osteoporosis is just one piece of a much larger puzzle. Other ageing‐related conditions, such as cardiovascular disease, which manifests differently in women with a higher burden of symptoms, greater comorbidities, and worse outcomes compared to men (Cenko et al., 2023; Taqueti, 2018), and neurodegenerative diseases like Alzheimer's disease, which disproportionately affect women (Boccalini et al., 2025; Sundermann et al., 2020), are often treated as generic ageing diseases, overlooking sex‐specific pathophysiology and biological triggers.

Meanwhile, AI and big data have been hailed as the future of personalised medicine, yet their impact on women's health remains limited by the lack of female‐specific datasets. Without comprehensive, sex‐specific data, AI‐driven models risk perpetuating existing biases rather than correcting them. Adding to this complexity, recent evidence highlights the role of environmental factors, that is, the exposome, including dietary exposures, pollutants and social interactions, in shaping ageing and age‐related disease risk (Argentieri et al., 2025; Woods et al., 2025). Truly advancing women's health at a personalised level requires a new framework that integrates systems‐level medicine, molecular mechanisms and environmental influences. Such a framework would, in turn, maximise the utility of AI by providing a structured, mechanistic context for its application. To use an analogy, relying on AI without such a framework is like giving it only a chessboard and a handful of pieces and asking it to play a game. There is information, but no organising logic. Providing a framework is equivalent to giving the AI the ‘rules of the game’: despite limited prior datasets, it can extrapolate how events may unfold and produce far more accurate, testable predictions.

The recently proposed Blueprint Theory (BPT) (Kern, Faragher, Woods et al., 2024) offers such a framework, that is, the ‘rules of the game’. BPT reframes ageing not as a continuous accumulation of random damage, but as the result of undesirable, unregulated triggering of biological pathways, termed ‘patho‐pathways’, driven by intrinsic factors (e.g., hormonal) and extrinsic factors (e.g., environmental) that shape age‐related phenotypes and the progression to chronic disease (Kern, Bonventre et al., 2025; Kern, Faragher, Woods et al., 2024; Woods et al., 2025). By systematically mapping these patho‐pathways through the creation of ‘Blueprint Maps’, BPT enables a more personalised understanding of ageing – particularly relevant for women, whose ageing trajectories are especially distinct and underexplored. Importantly, the framework provides a tool not only for identifying where things go wrong, but also for designing targeted interventions aimed at restoring biological equilibrium rather than merely suppressing symptoms. By conceptualising patho‐pathways as the maladaptive execution of biological pathways arising within interacting biological systems, BPT may be particularly informative during major physiological transitions such as menopause, when hormonal change can alter multiple interconnected processes simultaneously. This mechanistic scaffold may also help reconcile apparently contradictory clinical observations and enable AI models to generate biologically grounded, testable predictions even where existing datasets are incomplete.

## 2 THE CHALLENGE OF UNDERSTANDING AGEING IN WOMEN

Women experience unique health challenges, especially during and after menopause, which increase the risk of osteoporosis, metabolic dysfunction‐associated steatotic liver disease (MASLD; formerly NAFLD)(Kanwal et al., 2024), vascular calcification, kidney disease, type II diabetes (T2D), neurodegenerative diseases, endometrial hyperplasia and associated uterine cancer, and cardiovascular disease, amongst others (Epplein et al., 2008; Hannemann et al., 2007; Jeong & Park, 2022; Ji & Yu, 2015; Kamińska et al., 2023; Lee et al., 2012; Ottarsdottir et al., 2022). Environmental factors – such as toxin exposure, diet and lifestyle – further shape ageing and disease onset and progression trajectories, but are rarely integrated into research models (Woods et al., 2025).

AI offers a powerful tool for drug discovery and therapeutic development, but it performs best in domains where large, consistent and complete datasets exist and where the underlying structure of the problem is well defined. AlphaFold (Jumper et al., 2021) is a good illustration: it succeeds because the rules governing protein folding are fixed, and the target, that is, predicting a protein's three‐dimensional molecular structure from its sequence, is informed by decades of high‐quality experimental data generated through techniques such as X‐ray crystallography and nuclear magnetic resonance (NMR).

Ageing, by contrast, offers none of this clarity. Datasets are incomplete, small and inconsistent, and the underlying mechanisms are diffuse, multifactorial and only partially mapped. These challenges are even more pronounced in women's health. Data on women are particularly scarce, and the problem is compounded by physiological plasticity driven by shifting hormonal states, such as menopause, along with additional unresolved mechanisms. Together, these factors lead to incomplete or inaccurate models of ageing and age‐related disease. The added influence of individual environmental exposures further underscores the need for personalised medicine – an approach that remains out of reach without comprehensive, person‐specific data (Argentieri et al., 2025; Kern, Faragher, Woods et al., 2024).

Bayesian and other probabilistic AI strategies can help bridge these gaps by integrating prior knowledge from existing physiological and systems‐level frameworks, reducing dependence on large, specialised datasets. The central challenge, however, remains the development of a robust systems‐level framework capable of capturing the full complexity of ageing and its associated diseases.

## 3 THE BLUEPRINT THEORY: A FRAMEWORK FOR UNDERSTANDING AGEING

Despite widespread recognition that human biology operates as an interconnected system, modern medicine continues to treat diseases, such as cardiovascular disease, T2D, kidney disease and neurodegeneration, in silos. This fragmented approach contributes to the staggering 90% failure rate in clinical drug development (Sun et al., 2022). For women, this is particularly evident in the interconnected conditions triggered by menopause, such as osteoporosis, MASLD, neurodegeneration, T2D, kidney disease and cardiovascular disease.

One reason for this blind spot is the absence of a unifying framework to explain how diseases are mechanistically linked – whether through cumulative damage, feedback loops or environmental triggers. BPT aims to close this gap by reframing disease not as isolated events, but as interconnected phenomena driven by shared molecular mechanisms.

At the heart of BPT is the concept of ‘patho‐pathways’, that is, normally adaptive biological pathways that, when triggered out of context by intrinsic or environmental factors, drive pathological changes such as atrophy, hyperplasia, dysplasia and fibrosis. Resulting cellular changes underpin both visible ageing traits (e.g., sarcopenia, skin wrinkling, arthritis) and life‐limiting conditions such as cancer, Alzheimer's and cardiovascular disease.

BPT offers a practical framework for tracing and mapping how patho‐pathways are triggered by factors such as genetics, diet, stress, infections and pollution, and how they converge to accelerate ageing and disease (Figure 1). By tracing these mechanisms, BPT enables the creation of ‘blueprint maps’ that reveal shared vulnerabilities across diseases and identify core biological nodes for therapeutic intervention.

**FIGURE 1:**
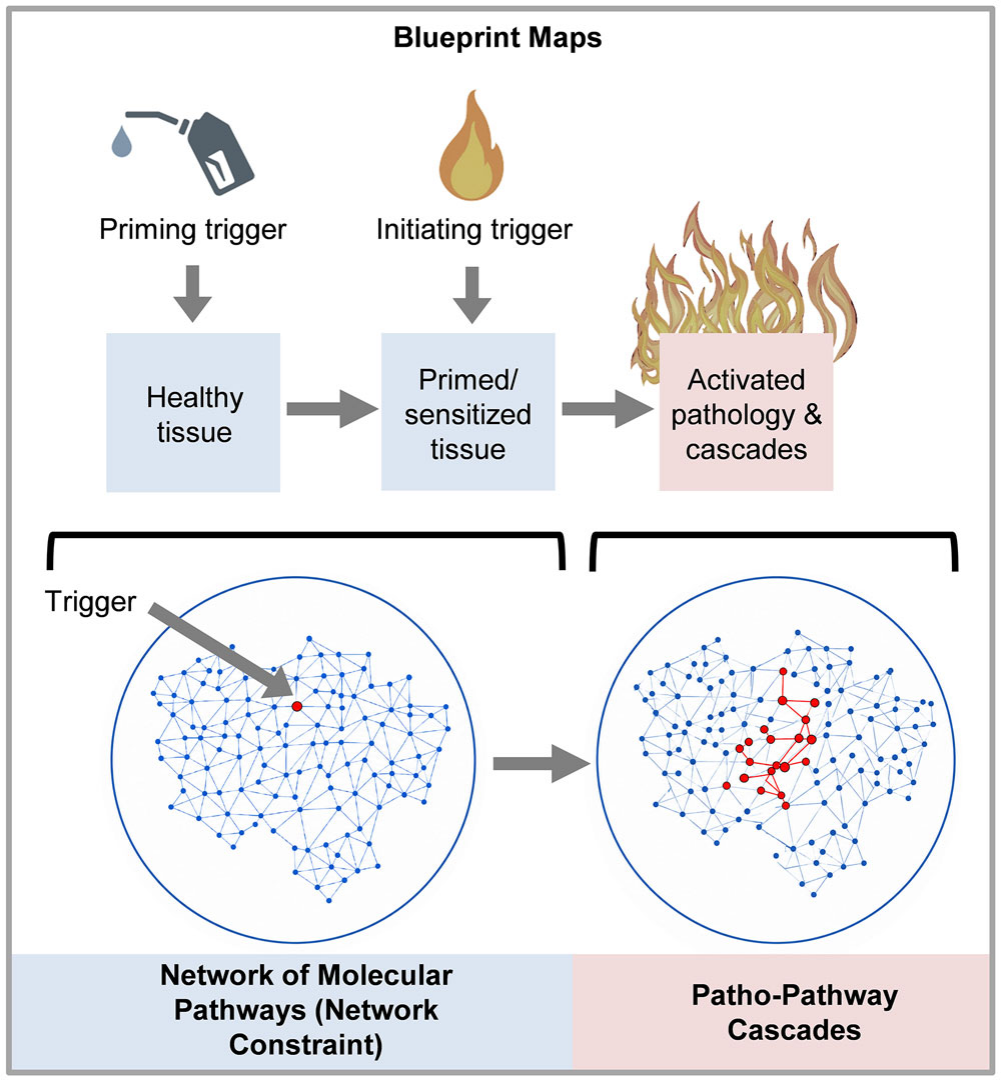
**Blueprint Theory framework illustrating the emergence and propagation of pathological pathways (patho‐pathways)**. The Blueprint Theory (BPT) conceptualises ageing as a transition from healthy tissue homeostasis to the maladaptive activation of biological pathways (‘patho‐pathways’) driven by intrinsic and extrinsic stressors. In the healthy baseline state (left), biological systems remain resilient and adaptive despite minor perturbations. With ageing, hormonal change, chronic inflammation, metabolic stress, environmental exposures and other stressors progressively prime or sensitise tissues (centre), lowering the threshold for patho‐pathway activation. Once initiated, a single patho‐pathway can trigger a domino‐style knock‐on effect, leading to cascades of interconnected patho‐pathways that propagate dysfunction across tissues and organ systems. In women, menopause‐associated oestrogen decline is proposed to represent a major priming event that predisposes inflammatory and metabolic patho‐pathways, contributing to osteoporosis, vascular calcification, metabolic dysfunction‐associated steatotic liver disease (MASLD), type 2 diabetes, cardiovascular disease, kidney disease and neurodegeneration. BPT reframes these conditions not as isolated diseases but as interconnected manifestations of shared patho‐pathways. Figure created partially under license in BioRender and partially using images under license from iStock.com.

A major insight of BPT is that many diseases are not separate entities but share a small number of common pathological drivers, that is, key patho‐pathways. Much like critical nodes in an electrical grid, failure at these points can cause widespread system dysfunction. By targeting these nodes, that is, key patho‐pathways, BPT opens the door to multi‐disease therapies that could dramatically improve outcomes and reduce healthcare inefficiencies.

## 4 TESTABLE PREDICTIONS

One key benefit of BPT is that it generates testable, real‐world predictions. For example, it anticipated the broad, system‐level effects of therapeutics like glucagon‐like peptide‐1 (GLP‐1) receptor agonists – including semaglutide (Ozempic, Rybelsus, Wegovy), dulaglutide (Trulicity) and tirzepatide (Mounjaro, Zepbound) – as early as 2023 (Kern, Faragher, Bonventre et al., 2024; Kern, Faragher, Woods et al., 2024), based on the identification of GLP‐1 signalling as a key patho‐pathway (Kern, Jenkins et al., 2025). Indeed, the effects of these drugs appear to extend far beyond obesity and type 2 diabetes. Research emerging over the past few years suggests that GLP‐1 receptor agonists may also play important roles in the treatment of cardiovascular, kidney and liver diseases, as well as neurodegeneration, and potentially even ageing itself (Jumper et al., 2021; Ryan et al., 2024; Zheng et al., 2024).

As a second example, BPT identified another key intervention node with the potential to exert therapeutic effects across multiple diseases simultaneously. In other words, a ‘Swiss Army knife’ therapeutic target (Figure 2). Specifically, it highlighted a necrosis (unregulated cell death) patho‐pathway as a shared pathological driver spanning kidney disease, cardiovascular disease (including myocardial infarction and stroke), liver disease, cancer, neurodegeneration and ageing more broadly (Kern, Bonventre et al., 2025). Importantly, targeting this necrosis patho‐pathway has the potential to minimise adverse trade‐offs, as this intervention acts on an unprogrammed process that is not under genetic regulation, thereby reducing the likelihood of deleterious side effects (Kern, Bonventre et al., 2025; Kern, Jenkins et al., 2025).

**FIGURE 2:**
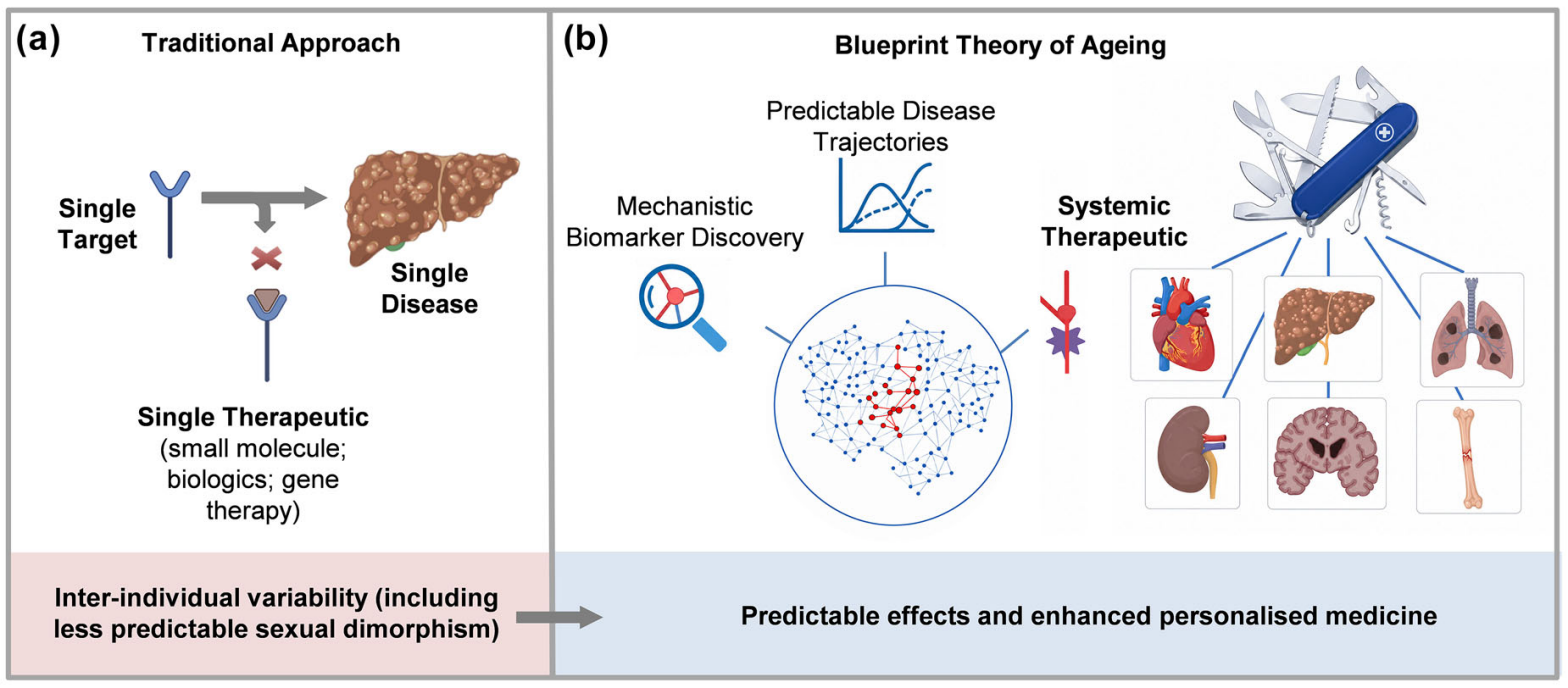
**Systems‐level implications of the Blueprint Theory for personalised medicine and women's health**. (a) Conventional approaches treat age‐related diseases as isolated entities, resulting in fragmented therapeutic strategies with limited predictive power and substantial inter‐individual variability. These limitations are particularly evident in women's health, where historical under‐representation in biomedical research and the physiological changes associated with menopause contribute to greater variability in disease presentation, therapeutic response and clinical outcomes. (b) The Blueprint Theory (BPT) proposes integrative blueprint maps that identify shared pathological pathways (‘patho‐pathways’) linking diseases across organ systems. By revealing common mechanistic drivers and interconnected patho‐pathway networks, BPT enables the identification of key intervention nodes, improving prediction of disease onset, progression and therapeutic response, while facilitating the discovery of mechanism‐based biomarkers and system‐level therapeutics. Rather than targeting individual diseases, these intervention nodes provide opportunities to address multimorbidity through ‘Swiss Army knife’ therapeutic targets with potential applicability across multiple age‐related diseases. Figure created partially under license in BioRender and partially using images under license from iStock.com.

## 5 RESOLVING MEDICAL PARADOXES: UNRAVELLING THE CALCIUM PARADOX

BPT not only offers a framework for identifying therapeutic targets; a second benefit of the BPT is that it helps untangle some of the most enduring medical puzzles. One such example is the calcium paradox observed where osteoporosis (calcium loss from bone) and arterial calcification co‐occur. This is even in the presence of completely normal circulating calcium levels, underscoring that the problem lies in regulation, not supply (Persy et al., 2006). The calcium paradox is observed in both men and women, but it is far more pronounced, earlier and more clinically impactful in women, especially during and after menopause.

First described in the 1990s (Persy et al., 2006), this paradox highlights the simultaneous occurrence of osteoporosis and arterial calcification. Essentially, the body is running ‘build bone’ and ‘break bone’ pathways at the wrong time, in the wrong tissues.

What is significant to note is that it is not simply a case that blood calcium (derived from bone loss) has no outlet and therefore builds up in the vasculature. During menopause, a lack of parathyroid hormone (PTH) and parathyroid hormone‐related protein (PTHrP) allows for the excretion of excessive calcium through urine, preventing a build‐up of calcium (that is, calcium wasting occurs) (Kovacs, 2016; Pollycove et al., 2011).

BPT offers a potential explanation for the calcium paradox in the form of a testable, ‘risky’ or Popperian prediction. It posits that a key inflammatory patho‐pathway – triggered by factors such as obesity, inflammaging, and especially in women, menopause due to oestrogen decline following follicular atresia – acts differently on bone and vasculature. This pathway alone is sufficient to induce bone loss, but vascular calcification requires additional priming factors, such as elevated glucose and lipid levels, which make vascular tissue susceptible to a secondary patho‐pathway that drives ectopic calcification through the pathological transdifferentiation of vascular cells into bone‐like cells (that is, pseudo‐osteoblasts) (Abedin et al., 2004; Chen & Moe, 2012; Demer, 2002; Kern, Faragher, Woods et al., 2024; Virchow, 1863). In other words, while a single inflammatory patho‐pathway can cause osteoporosis, vascular calcification manifests only in tissue preconditioned by metabolic stress.

This reveals that the two conditions are not independent diseases, as traditionally assumed, but different manifestations of a shared underlying mechanism with tissue‐specific thresholds. That is to say, rather than representing entirely separate pathologies, osteoporosis and vascular calcification share a common inflammatory patho‐pathway, with tissue‐specific susceptibility – particularly pronounced in women – determining their distinct outcomes (Kern, Faragher, Woods et al., 2024).

Notably, rather than being viewed as separate diseases, osteoporosis and vascular calcification, they can be classified according to the common inflammatory patho‐pathway they share, enabling a paradigm shift toward a more systemic approach to medicine (Kern, Jenkins et al., 2025).

The value added by BPT lies in providing the first cohesive systems‐biology framework capable of reconciling multiple unexplained physiological observations – normal serum calcium, bone loss, vascular calcification, metabolic stress and menopausal changes – under one mechanistic model. Under BPT, the calcium paradox becomes far less paradoxical. Rather than treating bone and artery as independent targets of calcium imbalance, BPT demonstrates that they are functionally coupled through inflammatory and metabolic stress, which activates inappropriate cellular pathways (e.g., osteoclast overactivity and vascular transdifferentiation).

Resolving this paradox offers three key advantages (Figure 2):

1. *Greater disease predictability* of onset and progression.
2. *Improved biomarker identification*, through mechanistic understanding rather than reliance on trial‐and‐error correlations.
3. *Enhanced therapeutic development*, supported by mechanistic insight into shared key pathological pathways (patho‐pathways).

## 6 EXPLAINING DISEASE SYNDROMES: MASLD, T2D, CARCINOMA AND NEURODEGENERATION

A framework such as the BPT can help elucidate the complex interconnections within disease syndromes, including obesity‐related metabolic syndrome, or menopause‐related syndromes that disproportionately affect women. One example is metabolic dysfunction‐associated steatotic liver disease (MASLD), which develops through insulin resistance‐driven patho‐pathways initiated by lipotoxicity, namely the excessive uptake of lipids by non‐adipose tissues. Lipotoxicity arises from the release of free fatty acids (FFAs) from adipose tissue under several conditions: when adipose tissue reaches its storage capacity, leading to local hypoxia and insulin resistance, as observed in obesity; through inflammation‐induced adipocyte insulin resistance, as seen in inflammaging; or as a consequence of menopause, where oestrogen decline promotes excessive and dysregulated FFA release. Collectively, these conditions converge on broadly similar underlying patho‐pathways, although they are triggered by distinct biological drivers depending on the syndrome.

The process proceeds as follows:

1. FFAs are taken up by non‐adipose organs, such as the liver, causing lipotoxicity and localised insulin resistance patho‐pathways.
2. These localised patho‐pathways promote further lipid accumulation and associated lipotoxicity in organs such as the liver, alongside disruption of glucose metabolism, thereby creating a positive feedback loop that drives systemic insulin resistance, metabolic syndrome and related conditions such as MASH and T2D (Adams et al., 2005; Kern, Faragher, Woods et al., 2024; Sears & Perry, 2015; Unger, 2003).

This is particularly relevant in post‐menopausal women, whose hormonal shifts exacerbate visceral fat accumulation, insulin resistance and chronic inflammation, overall placing them at higher risk for MASLD and its complications. Hepatic insulin resistance disrupts blood glucose control by promoting glucose secretion while reducing muscle uptake, further aggravating T2D. Over time, MASLD can progress to metabolic dysfunction‐associated steatohepatitis (MASH; formerly non‐alcoholic steatohepatitis, NASH) (Kanwal et al., 2024), a more aggressive form characterised by severe inflammation and hyperactive wound‐healing responses that drive fibrosis, cirrhosis, and increased risk of hepatocellular necrosis and associated carcinoma (Adams et al., 2005; Kanwal et al., 2024; Kern, Bonventre et al., 2025).

Chronic inflammation from conditions like MASLD can also promote neurodegeneration via additional inflammation patho‐pathway activation, compounded in women by potentially greater reductions in cerebral blood flow in later life (Marley et al., 2020). Systemic inflammatory patho‐pathways propagate to reach the brain through multiple routes – including the blood–brain barrier, vascular activation and neural pathways such as the vagus nerve (Xie et al., 2021). For instance, neuroinflammation exacerbates Alzheimer's disease through microglial hyperactivation, worsening amyloid‐beta and tau pathologies and contributing to cognitive decline (Heppner et al., 2015; Ising et al., 2019; Sala Frigerio et al., 2019). Similarly, Parkinson's disease is influenced by systemic inflammation and neuroinflammatory triggering (Wang et al., 2015).

The new value that BPT adds is its ability to connect these traditionally separate conditions, such as MASLD, T2D, carcinoma and neurodegeneration, into one coherent mechanistic model. Rather than viewing these diseases as unrelated comorbidities, BPT demonstrates that they are linked through shared patho‐pathways (e.g., inflammation, insulin resistance) whose activation thresholds differ across tissues and are especially modulated by menopausal physiology in women. This systems‐level perspective reveals previously unrecognised causal relationships – such as how metabolic stress primes certain tissues for secondary patho‐pathways – offering a unified explanation for patterns of multimorbidity that have long appeared disconnected (Figure 1).

By mapping these interconnections, BPT allows researchers to trace how key patho‐pathways, such as inflammation patho‐pathways and insulin resistance patho‐pathways, ripple across multiple organ systems. This framework facilitates the identification of shared pathological drivers and potential therapeutic targets across age‐related diseases, providing a personalised, systems‐level approach that is particularly valuable for women navigating complex physiological transitions like menopause.

## 7 THERAPEUTIC IMPLICATIONS OF BPT

A framework like the BPT provides not only a model for mapping the molecular pathways of age‐related disease and degenerative change but also introduces a mechanistic logic that enables far more precise therapeutic strategies – ones grounded in how patho‐pathways actually emerge and interact across systems. This represents a shift from treating downstream symptoms to targeting upstream drivers with predictable, testable effects.

One of the key challenges in therapeutic development, including those geared toward treating ageing‐related conditions, is finding ways to disrupt pathological feedback loops as those detailed above, without causing further harm or unintended consequences. That is to say, the goal is to restore normal physiology without falling into the trap of creating trade‐off consequences or pushing biological systems too far in one direction, that is, causing overcorrection. This is particularly problematic for long‐duration treatment therapies.

For example, rapamycin, a drug known for its ability to extend lifespan in various animal models, works by inhibiting the mechanistic target of rapamycin pathway (mTOR), which is involved in cell growth and metabolism. In humans, rapamycin, by dampening bone remodelling, can protect against osteoporosis (a bone loss disorder); however, it is also deleterious to fracture healing (Holstein et al., 2008; Shen et al., 2018). As another example, GLP‐1 inhibitors bring about significant health improvements by reducing body fat and metabolic syndrome, but the same depletion of facial fat comes at the cost of compromised skin, including wrinkles and sagging (‘Wegovy face’ or ‘Ozempic face’) (Humphrey & Lawrence, 2023).

What BPT newly offers is a mechanistic basis for understanding and therefore avoiding these trade‐offs: by identifying the exact patho‐pathways driving each condition, BPT makes it possible to identify and target the specific nodes that generate disease while sparing the pathways necessary for healthy function. This fine‐tuning is only possible when physiology is viewed holistically and mechanistically, rather than as isolated organ‐specific disorders.

BPT's unified model linking hormone shifts, inflammation, metabolic stress and tissue‐specific susceptibility provides a roadmap for designing therapies that minimise collateral damage by intervening at the correct mechanistic leverage points. This allows therapeutic design to move beyond merely ‘broad‐acting’ interventions (which frequently cause side effects) toward targeted, personalised strategies that can break pathological loops without destabilising healthy biological processes.

In this way, BPT holds promise for developing interventions that reduce unintended consequences, personalise therapeutic targeting and ultimately restore physiological balance with far greater precision than current approaches allow.

## 8 CONCLUSION

To realise the promise of personalised longevity medicine for men and women, we must rebuild its foundation around three pillars:

1. AI trained on robust, complete and sex‐specific datasets.
2. Genomic and molecular tools that capture biological heterogeneity.
3. Environmental (i.e., exposomic) insights reflecting real‐world ageing.

Above all, these pillars require a unifying framework capable of integrating complex, interconnected biological processes. BPT provides precisely such a model. By mapping shared patho‐pathways and their systemic interactions, BPT enables a coherent, mechanistic understanding of ageing and age‐related disease. It allows researchers to link seemingly distinct and yet interconnected conditions, such as metabolic dysfunction and neurodegeneration, through a single, actionable framework, guiding both biomarker discovery and therapeutic development.

Importantly, BPT also resolves longstanding medical paradoxes, such as the calcium paradox, by revealing how shared inflammatory and metabolic patho‐pathways can manifest differently across tissues. The framework delivers the multiple tangible benefits (Figure 2), including greater predictability of disease onset and progression, improved biomarker identification and enhanced therapeutic development.

Furthermore, BPT enables the effective use of incomplete or sparse datasets, especially when it comes to scarce datasets around women's health, providing a structured context for AI and other computational tools to make accurate predictions despite gaps in data. The future of healthspan and lifespan must be built around physiological heterogeneity by design, and frameworks like BPT are essential for achieving truly personalised, systems‐level medicine.

## AUTHOR CONTRIBUTIONS

Carina C. Kern conceptualised and wrote the first draft, after which Rosie Richardson, Tina Woods, Nikodem Grzesiak and Annalisa Jenkins contributed equally to the development and revision of the manuscript. All authors contributed equally to the manuscript. All authors have read and approved the final version of this manuscript and agree to be accountable for all aspects of the work. All persons designated as authors qualify for authorship, and all those who qualify for authorship are listed.

## CONFLICT OF INTEREST

Carina C. Kern, Nikodem Grzesiak and Rosie Richardson serve as CEO, CTO and Senior Scientist at LinkGevity, respectively. Annalisa Jenkins is Chair of The Court of the London School of Hygiene & Tropical Medicine and sits on the boards and advisory boards of several private and public companies, including Genomics England and LinkGevity, and was formerly committee member of the Science Board to the US Food & Drug Administration. She is a trustee of the British Heart Foundation and Cancer Research Horizons at Cancer Research UK. Tina Woods is CEO of Collider Health and trustee of the British Society for Research on Aging.

## FUNDING INFORMATION

None.

## GENERATIVE AI STATEMENT

Generative AI assistance (OpenAI ChatGPT) was used to check and improve the grammar, language, readability and clarity of the manuscript. All scientific content, interpretations and conclusions were developed and verified by the authors, who take full responsibility for the content of the publication.
